# Threshold-Effect Association of Dietary Cholesterol Intake with Dyslipidemia in Chinese Adults: Results from the China Health and Nutrition Survey in 2015

**DOI:** 10.3390/nu11122885

**Published:** 2019-11-27

**Authors:** Qiumin Huang, Hongru Jiang, Bing Zhang, Huijun Wang, Xiaofang Jia, Feifei Huang, Liusen Wang, Zhihong Wang

**Affiliations:** National Institute for Nutrition and Health, Chinese Center for Disease Control and Prevention, 29 Nanwei Road, Beijing 100050, China; qmhuangx@163.com (Q.H.); jhr_seven@163.com (H.J.); zzhangb327@aliyun.com (B.Z.); wanghj@ninh.chinacdc.cn (H.W.); jiaxf@ninh.chinacdc.cn (X.J.); fayekobe@163.com (F.H.); wangliusen1993@163.com (L.W.)

**Keywords:** dietary cholesterol, dyslipidemia, hypercholesterolemia, LDL-hypercholesterolemia, HDL-hypocholesterolemia, hypertriglyceridemia, Chinese adults

## Abstract

The association of dietary cholesterol intake with dyslipidemia and subtypes is controversial. This study aimed to examine the association of dietary cholesterol intake with dyslipidemia and subtypes in Chinese adults. Using data from the China Health and Nutrition Survey (CHNS) in 2015, the present study selected 4383 participants aged 18–59 years who were free of diabetes, apoplexy, and myocardial infarction disease. Information was obtained on dietary intake, anthropometric measurements, and blood laboratory measurements. Dietary cholesterol intake was calculated based on the data collected by consecutive 3 days 24 h recalls combined with the weighing of household seasonings and categorized by 11 levels: The first 10 levels in ranges of 50 mg/day and the 11th level at ≥500 mg/day. Dyslipidemia, hypercholesterolemia, hypertriglyceridemia, low-density lipoprotein (LDL)-hypercholesterolemia, and high-density lipoprotein (HDL)-hypocholesterolemia were defined based on the Chinese adult dyslipidemia prevention guide (2016 edition). Multivariable logistic regressions were performed to examine the association of dietary cholesterol intake levels with dyslipidemia and subtypes. The prevalence of dyslipidemia was 37.5% among Chinese adults in 2015 (hypercholesterolemia 9.6%, HDL-hypocholesterolemia 21.1%, LDL-hypercholesterolemia 12.7%, and hypertriglyceridemia 15.2%). The lowest prevalence of hypercholesterolemia and LDL-hypercholesterolemia was 6.7% and 9.4%, respectively, which was relative to a dietary cholesterol intake level of 100.0 to <150.0 mg/day. After adjusting for all potential confounders, adults with the highest dietary cholesterol intake level of ≥500 mg/day compared with the dietary cholesterol intake of 100.0 to <150.0 mg/day showed one-time higher odds of hypercholesterolemia (odds ratios (OR) 2.0, 95% confidence intervals (CI) 1.3–3.3), as well as LDL-hypercholesterolemia (OR 2.0, 95% CI 1.3–3.0), but a null association of dietary cholesterol intake with dyslipidemia, hypertriglyceridemia, and HDL-hypocholesterolemia. The study suggested that a dietary cholesterol intake level of 500 mg/day and above may be a threshold point for high odds of hypercholesterolemia and LDL-hypercholesterolemia.

## 1. Introduction

Dyslipidemia is abnormal lipid metabolism characterized by any one of hypercholesterolemia, high-density lipoprotein (HDL)-hypocholesterolemia, low-density lipoprotein (LDL)-hypercholesterolemia, and hypertriglyceridemia [1]. Many studies suggested the key role of controlling dyslipidemia in the prevention of cardiovascular disease [2,3,4], the leading cause of mortality in China [5]. It is, therefore, crucial to identify modifiable risk factors, like dietary intake, to control the epidemic of dyslipidemia and related disease burden in China.

The association of dietary cholesterol intake with dyslipidemia has been gaining a high level of concern due to inconsistent evidence across studies. In 2017, Mente et al. reported that dietary cholesterol intake was associated with an increase in both serum total cholesterol (TC) and LDL-C and also with the increased LDL-C to HDL-C ratio [6]. A meta-analysis study of 17 randomized control trial studies conducted from 1974 to 1999 revealed that the addition of 100 mg of dietary cholesterol increased the total/HDL cholesterol ratio [7]. And results from the controlled feeding studies showed changes in serum cholesterol ranging from 2.2 to 4.5 mg/dL per 100 mg/day of change in dietary cholesterol [8]. On the contrary, others have shown a null association [9,10], because investigators have reported that increased intake of dietary cholesterol (exogenous) was associated with the decreased synthesis of endogenous de novo cholesterol, possibly as a compensatory mechanism that keeps cholesterol homeostasis constant [11,12]. In China, one study using China Health and Nutrition Survey (CHNS) 2009 found that the adults with a high intake of dietary cholesterol (≥300.0 mg/day) had higher odds of hypercholesterolemia than the adults with intake of <300 mg/day [13]. Another study using the Shanghai diet and health survey (SDHS) showed 60% higher odds of dyslipidemia prevalence among Shanghai adults with a top quintile of dietary cholesterol intake (≥538.0 mg/day) as compared to the odds with a bottom quintile (<193.1 mg/day) [14].

Although both Chinese dietary guidelines and the dietary guidelines for Americans eliminated the recommendation of no more than 300.0 mg/day of dietary cholesterol intake [15,16]. One study showed that Chinese adults in higher quantile had a much bigger increase in dietary cholesterol intake from 1991 to 2011 as compared with those in lower quantile. The current evidence does not seem enough to avoid any recommendation of dietary cholesterol intake, given the controversial results over studies [17]. To better understand their relationship, this study aimed to fully examine the dose-response association of dietary cholesterol intake, by refining its grouping, with prevalence of dyslipidemia and subtypes among Chinese adults aged 18–59 years using the data collected in CHNS 2015.

## 2. Subjects and Methods

### 2.1. Study Population

Data for this analysis were from the CHNS, established by the National Institute for Nutrition and Health, Chinese Center for Disease Control and Prevention, and the University of North Carolina at Chapel Hill. The CHNS is an ongoing series of longitudinal household surveys investigating how social and economic changes in China affect various nutrition- and health-related outcomes. The CHNS was conducted in 9 rounds across 9 provinces (autonomous regions) during the period of 1989–2009. The 9th round was conducted in 2011 across 12 provinces (autonomous regions) with the additions of Beijing, Shanghai, and Chongqing, and the 10th round was conducted in 2015 across 15 provinces (autonomous regions) with the additions of Shanxi, Yunnan, and Zhejiang. Multistage, random cluster sampling was used to select the survey sample in each province to ensure that urban and rural areas were represented in the CHNS. The survey design and methods have been described in detail elsewhere [18].

Our analysis used the 10th-round survey of CHNS in 2015. From all subjects aged 18–59 years who had complete socioeconomic status and demographic data, 3 days 24 h dietary recall data, and biochemical data, excluding those with diabetes, apoplexy, and myocardial infarction disease, those with implausible energy intakes (<800 kcal/day or >6000 kcal/ day for men and <600 kcal/day or >4000 kcal/day for women), and those with missing dietary cholesterol intake and serum lipids. Subjects with diabetes, stroke, and myocardial infarction disease were defined as having hospital records diagnosed by professional doctors or receiving treatment for these diseases. A total of 4383 adults (1949 males and 2434 females) were included in the final analyses. This study was reviewed and approved by the institutional review board of the University of North Carolina at Chapel Hill and the Chinese Center for Disease Control and Prevention. Written informed consent was obtained from all subjects.

### 2.2. Assessment of Dietary Cholesterol Intake

CHNS 2015 collected dietary data using 3 consecutive 24 h recalls (2 weekdays and 1 weekend day) for each individual and weighed seasonings in the household inventory over the same period. Trained health workers interviewed the participants on each of those days to collect all food consumption (type, amounts, type of meal, and place of consumption) at home and away from home during the preceding 24 h. Individuals’ consumption of seasonings was calculated according to eating times at home and individuals’ energy proportion among family members. Details of the collection method are described elsewhere [18,19]. The individual daily intake of dietary energy, macronutrients, cholesterol, and micronutrients was calculated according to daily food and condiments consumption using the China Food Composition Table [19]. Dietary cholesterol intake levels were categorized as <50 mg/day, 50 to <100 mg/day, 100 to <150 mg/day, 150 to <200 mg/day, 200 to <250 mg/day, 250 to <300 mg/day, 300 to <350 mg/day, 350 to <400 mg/day, 400 to <450 mg/day, 450 to <500 mg/day, and ≥500 mg/day.

### 2.3. Definitions of Dyslipidemia

For lipids detection, a fasting blood sample was collected by experienced physicians, phlebotomists, or nurses via venipuncture. Plasma and serum samples were frozen and stored at −80 °C for later laboratory analysis. Blood samples were analyzed at the laboratory of the National Institute for Nutrition and Health. Serum concentrations of triglycerides (TG) and total cholesterol (TC) were measured using the glycerol-phosphate oxidase method, and low-density lipoprotein cholesterol (LDL-C) and high-density lipoprotein cholesterol (HDL-C) were measured using the polyethylene glycol-modified enzyme method by determiner regents (Kyowa Medex Co. Ltd., Tokyo, Japan) on a Hitachi 7600 automated analyzer (Hitachi Inc., Tokyo, Japan).

According to the Chinese adult dyslipidemia prevention guide (2016 edition), serum TC concentrations greater than 6.2 mmol/L (240 mg/dL) were considered to be hypercholesterolemia, serum TG concentrations greater than 2.3 mmol/L (200 mg/dL) were considered to be hypertriglyceridemia, serum LDL-C concentrations greater than 4.1 mmol/L (160 mg/dL) were considered to be LDL-hypercholesterolemia, and serum HDL-C concentrations less than 1.0 mmol/L (40 mg/dL) were considered to be HDL-hypocholesterolemia [20]. Dyslipidemia was defined as having at least one of the following: Hypercholesterolemia, hypertriglyceridemia, LDL-hypercholesterolemia, or HDL-hypocholesterolemia.

### 2.4. Assessment of Covariates

Information on sociodemographic (e.g., gender, age, geographic region, education, and income) and lifestyle (e.g., smoking and drinking status and physical activity) variables were collected through a combination of self-administered and interviewer-administered questionnaires. All the involved provinces were grouped into 3 major economic zones (central, east, and west) based on China’s “Seventh Five-Year Plan”. The west region included the city of Chongqing and the provinces of Guizhou, Yunnan, Shanxi, and Guangxi. The central region included the provinces of Heilongjiang, Henan, Hubei, and Hunan. The east region included the cities of Beijing and Shanghai and the provinces of Jiangsu, Zhejiang, Shandong, and Shanxi. Education was categorized as primary school or less, secondary school, and high school or more. The yearly income per capita was obtained by asking the head of the household and categorized into tertiles (low, medium, and high). Smoking and drinking statuses were categorized as current and current non-. Physical activity was assessed by metabolic equivalent-hours/week (MET-h/week), which equaled intensity of physical activity multiplied by physical activity time. The intensity of physical activity assignments referred to the American College of Sports Medicine Association’s recommended standard [21].

Weight and height were measured to the nearest 0.1 kg and 0.1 cm, respectively, with the participants in lightweight clothing and without shoes. Body mass index (BMI) was calculated from measured weight (kg) and height (m) squared (kg/m^2^) [22]. Three blood pressure (BP) measurements were taken using regularly calibrated mercury sphygmomanometers on the right arm with the participant in a seated position after at least 5 minutes of rest in a quiet room. Participants were advised to avoid cigarette smoking, alcohol, caffeinated beverages, and exercise for at least 30 minutes before the measurement. Systolic blood pressure (SBP) was measured at the first appearance of a pulse sound (Korotkoff Phase 1) and diastolic blood pressure (DBP) at the disappearance of the pulse sound (Korotkoff Phase 5). Hypertension was defined as having SBP ≥140 mm Hg and/or DBP ≥90 mm Hg according to the mean of three BP measurements or using antihypertensive medications.

### 2.5. Statistical Analysis

Firstly, statistical interaction tests between dietary cholesterol intake and gender, age, and BMI were performed, and no statistical significance was found. The median value for continuous variables was presented, given the non-normal distribution tested by Kolmogorov-Smirnov and percentage of the total for categorical variables. Differences among daily dietary cholesterol intake levels were examined using the Wilcoxon rank-sum and Kruskal–Wallis H test for continuous variables and Chi-square test for categorical variables. A Cochran–Armitage test for trend was conducted to test the trend for prevalence of dyslipidemia, hypercholesterolemia, hypertriglyceridemia, HDL-hypocholesterolemia, and LDL-hypercholesterolemia with the increase of dietary cholesterol intake.

A series of multivariate binomial logistic regression models were conducted to assess the odds ratios (OR) and 95% confidence intervals (CI) by levels of dietary cholesterol intake, adjusting for potential confounders, including demographics, socioeconomic status, lifestyle, related intake of food groups and nutrients, and BMI value, in turn considering no multicollinearity. In addition, the linear trends were tested by assigning median values to levels of dietary cholesterol intake, and the variable modeled as a continuous term.

All statistical analyses were performed using SAS version 9.4 software (SAS Institute Inc., Cary, NC, USA). Formal hypothesis testing was two-sided with a significance level of 0.05.

## 3. Results

### 3.1. Baseline Characteristics

The baseline characteristics of participants across levels of dietary cholesterol intake are summarized in Table 1. Of all participants, the daily dietary cholesterol intakes for gender, age group, geographic region, educational level, yearly income, drinking status, hypertension history, and dietary nutrient intakes were statistically significant (*p* < 0.05). Participants with the highest daily dietary cholesterol level consumed more dietary intakes of energy, carbohydrate, protein, fat, fiber, vitamin C, vitamin E, niacin, and selenium (*p* < 0.001).

### 3.2. Prevalence of Dyslipidemia Dyslipidemia, Hypercholesterolemia, Hypertriglyceridemia, HDL-Hypocholesterolemia, and LDL-Hypercholesterolemia in Participants

Of all participants, 37.5% had dyslipidemia, 9.6% had hypercholesterolemia, 15.2% had hypertriglyceridemia, 21.1% had HDL-hypocholesterolemia, and 12.7% had LDL-hypercholesterolemia. The prevalence of dyslipidemia, hypercholesterolemia, and LDL-hypercholesterolemia was significant for different dietary cholesterol intake levels and showed an upward trend with the increase of dietary cholesterol intake levels (*p* < 0.05). However, the lowest prevalence of hypercholesterolemia and LDL-hypercholesterolemia was 6.7% and 9.4%, respectively, which was relative to a cholesterol intake of 100.0–150.0 mg/day. In contrast, the prevalence and trend of dyslipidemia, hypertriglyceridemia, and HDL-hypocholesterolemia did not differ among different dietary cholesterol intake levels (see Table 2).

### 3.3. Associations of Dietary Cholesterol Intake Levels with Dyslipidemia, Hypercholesterolemia, Hypertriglyceridemia, HDL-Hypocholesterolemia, and LDL-Hypercholesterolemia

Table 3 shows the ORs of dyslipidemia, hypercholesterolemia, hypertriglyceridemia, HDL-hypocholesterolemia, and LDL-hypercholesterolemia according to levels of dietary cholesterol intake in Chinese adults. After adjusting for potential confounders in each model, compared with the dietary cholesterol intake of 100.0 to <150.0 mg/day, adults consuming the dietary cholesterol intake of 50.0 to <100.0 mg/day were found to decrease their odds of hypercholesterolemia, and adults consuming the dietary cholesterol intake of 200.0 to <250.0 mg/day, of 250.0 to <300.0 mg/day, and of ≥500.0 mg/day showed significantly higher odds of hypercholesterolemia and LDL-hypercholesterolemia.

After adjusting for all potential confounders, adults with the highest intake of dietary cholesterol showed two times the odds of hypercholesterolemia (OR 2.0, 95% CI 1.3–3.3), as well as LDL-hypercholesterolemia (OR 2.0, 95% CI 1.3–3.0). A null association of dietary cholesterol intake was observed, with odds of hypertriglyceridemia and HDL-hypocholesterolemia. None of the linear trend tests were statistically significant between dietary cholesterol intake level and the odds of dyslipidemia, hypercholesterolemia, hypertriglyceridemia, HDL-hypocholesterolemia, and LDL-hypercholesterolemia in adults. 

## 4. Discussion

Using CHNS 2015, the present study examined the prevalence of dyslipidemia and its subtypes and the impact of dietary cholesterol intake levels on them. It was found that an estimated 37.5% of Chinese adults aged 18–59 years in our study had at least one type of dyslipidemia, which was higher than 34.0% of Chinese adults aged ≥18 years from a representative research of the China National Survey of Chronic Kidney Disease [23] and 16.8% of Korean adults aged 40–64 years from a cross-sectional analysis of the National Health Insurance Service-National Sample Cohort database [24]. Furthermore, our study showed that approximately 10% of adults have hypercholesterolemia, 15.2% hypertriglyceridemia, 12.7% LDL-hypercholesterolemia, and more than 20% HDL-hypocholesterolemia. Then, the prevalence of hypercholesterolemia, LDL-hypercholesterolemia, and HDL-hypocholesterolemia increased about 1%, 2%, and 6%, respectively from CHNS 2009 to the CHNS 2015, whereas the prevalence of hypertriglyceridemia decreased 8% [25].

The refining grouping of dietary cholesterol intake with the top-level at ≥500 mg/day makes it possible to fully explore its dose-response association with dyslipidemia and its subtypes. Previous studies suggested that adults with lower total cholesterol in the blood (≤160 mg/dL) have a higher mortality rate [26,27], and a large-scale observational study of residents aged 35–89 in Hiroshima and Nagasaki found a significant 64% lower risk of stroke in people with a median cholesterol intake of 624 mg/day compared to those with a median cholesterol intake of 152 mg/day [28]. Our study found the lowest prevalence of hypercholesterolemia and LDL-hypercholesterolemia at a cholesterol intake level of 100.0–150.0 mg/day, rather than at level of <100 mg/day.

Further, it was only observed that adults with the top level of dietary cholesterol intake had significantly higher odds of hypercholesterolemia as well as LDL-hypercholesterolemia as compared with those with 100.0–150.0 mg/day. And no significant linear trends between them was observed, which may indicate the potential threshold-effect association between them. One prospective study suggested the cholesterol intake of ≥747.0 mg/day associated with increased risk of ischemic heart disease and cancer in Japanese middle-aged men (45–68 years) living in Hawaii [29]. Previous studies, defining the grouping of dietary cholesterol intake as ≥300 mg/day and <300 mg/day, had limited ability to discover the potential association between dietary cholesterol intake and dyslipidemia and may underestimate the effect magnitude [13,30]. Some studies choosing the lowest dietary cholesterol intake as the reference group may conceal potential adverse impact on dyslipidemia or subtypes in relation to extremely low intake versus reasonable intake.

Our study has several limitations. First, our study assessed dietary intake by 3 days 24 h recalls, which may have a relatively limited correction for within-subject variation. It posed the risk of underestimating the dietary intakes, especially for episodically consumed foods as compared with nonconsecutive 24 h recalls. However, the average intake over three days can offer a relatively valid estimate of usual diet, as has been shown in earlier research using the CHNS [31]. Second, although a number of potential confounding factors were adjusted, we still cannot avoid the possibility of residual confounding. Third, there was no information on the use of cholesterol-lowering medication, which may underestimate the prevalence odds of dyslipidemia in relation to the dietary cholesterol intakes. Fourth, our study only identified apoplexy in general as exclusion criteria given the lack of information to distinguish cerebral infarction from cerebral bleeding. In addition, the cross-sectional nature of our study does not allow us to evaluate causal associations between dietary cholesterol and dyslipidemia risks. Prospective studies with large sample sizes would be required to further investigate this association.

## 5. Conclusions

In conclusion, this study indicates high prevalence of dyslipidemia dominating hypertriglyceridemia and HDL-hypocholesterolemia in Chinese adults and a dietary cholesterol intake level of 100–150 mg/day in relation to the lowest prevalence of hypercholesterolemia and LDL-hypercholesterolemia. A dietary cholesterol intake level of 500 mg/day and above may be a threshold point for high odds of hypercholesterolemia and LDL-hypercholesterolemia. Keeping daily dietary cholesterol intake within a reasonable range in a healthy eating pattern should be recommended. Findings from our study have implications for nutrition intervention targeting adults with relatively high levels of dietary cholesterol intake. Whether one limited intake of 500 mg/day should be recommended requires large-scale prospective cohort studies on dietary cholesterol intake-health outcomes.

## Figures and Tables

**Table 1:** Basic characteristics of the study population ^1^.

Characteristics	Total	<50.0 mg/day	50.0–<100.0 mg/day	100.0–<150.0 mg/day	150.0–<200.0 mg/day	200.0–<250.0 mg/day	250.0–<300.0 mg/day	300.0–<350.0 mg/day	350.0–<400.0 mg/day	400.0–<450.0 mg/day	450.0–<500.0 mg/day	≥500.0 mg/day	*p*-value
**Percent of all adults (%)**	100.0	10.0	9.5	11.7	11.2	11.1	9.7	8.3	7.5	5.1	3.4	12.6	
**Gender (%)**													<0.001
Male	44.5	37.8	39.9	42.5	41.2	41.6	44.7	44.8	50.0	49.8	52.7	52.4	
Female	55.5	62.2	60.1	57.5	58.8	58.4	55.3	55.2	50.0	50.2	47.3	47.6	
**Age (%)**													<0.001
18–44 years	41.4	31.2	43.5	40.3	44.9	38.9	47.3	46.4	42.4	39.5	38.7	41.3	
45–59 years	58.6	68.8	56.5	59.7	55.1	61.1	52.7	53.6	57.6	60.5	61.3	58.7	
**Geographic region**													<0.001
Central	35.9	43.6	35.6	35.6	40.4	35.6	33.9	31.3	33.0	39.5	36.0	31.2	
East	38.5	40.8	35.6	36.0	28.4	33.3	37.4	41.5	47.3	39.5	41.3	46.9	
West	25.7	15.6	28.9	28.4	31.2	31.1	28.7	27.2	19.7	21.1	22.7	21.9	
**Educational level**													
≤Primary school	21.2	35.1	19.7	24.3	21.8	21.8	20.9	16.8	16.4	16.6	14.7	17.0	<0.001
Secondary school	38.0	39.9	44.7	38.8	37.4	36.2	32.9	39.0	35.8	39.5	35.3	37.5	
≥High school	40.8	25.0	35.6	37.0	40.8	42.0	46.1	44.2	47.9	44.0	50.0	45.5	
**Yearly Income**													<0.001
Low	33.4	45.2	38.5	36.4	32.2	32.5	33.2	30.5	26.4	28.7	30.0	27.9	
Medium	33.3	31.2	35.6	32.3	33.3	36.0	32.5	30.8	38.2	36.3	32.7	30.4	
High	33.3	23.6	26.0	31.3	34.5	31.5	34.4	38.7	35.5	35.0	37.3	41.7	
BMI (kg/m^2^)	24.3	24.2	24.0	23.9	23.7	24.0	23.9	24.0	24.6	24.0	23.6	24.4	0.051
Smoking (%)	24.0	22.3	20.9	22.7	24.9	24.1	23.1	23.9	23.9	28.7	23.3	28.1	0.319
Drinking (%)	29.4	24.8	26.2	29.2	28.6	29.2	28.9	27.8	29.7	32.7	35.3	35.1	0.032
Hypertension (%)	26.3	33.7	21.6	25.2	25.1	24.5	27.1	22.5	26.4	29.6	24.7	28.8	0.006
Physical activity (MET-h/week)	30.3	32.5	29.0	20.0	21.6	37.8	32.0	42.8	30.6	26.4	28.3	35.5	0.253
**Dietary intakes**													
Energy (kcal/day)	1869.7	1650.5	1610.9	1723.4	1761.2	1781.5	1886.9	1922.6	2029.9	2085.8	2011.9	2286.0	<0.001
Carbohydrate (g/day)	230.4	239.7	211.3	224.2	218.3	218.8	229.5	228.1	244.8	237.1	244.0	253.3	<0.001
Fat (g/day)	70.7	46.7	58.7	65.1	68.1	74.8	69.1	76.3	78.1	82.3	76.3	91.8	<0.001
Protein (g/day)	58.4	44.6	46.9	50.9	53.0	55.6	61.2	63.2	68.6	68.0	71.8	84.2	<0.001
Fiber (g/day)	9.9	10.0	9.4	9.2	9.1	9.6	10.0	10.1	10.3	10.2	10.5	11.6	<0.001
Vitamin C (mg/day)	61.8	52.5	55.0	56.7	54.5	61.0	60.0	67.7	64.7	72.7	69.2	76.6	<0.001
Vitamin E (mg/day)	24.5	22.6	22.0	24.9	23.9	22.9	22.9	24.3	25.7	26.2	26.4	28.8	<0.001
Niacin (mg/day)	13.4	10.0	11.2	11.9	12.4	13.2	14.0	14.7	14.7	15.0	16.2	17.8	<0.001
Selenium (µg/day)	39.2	30.3	30.2	32.7	34.2	37.2	39.8	42.0	48.4	49.0	47.6	58.3	<0.001

Abbreviation: BMI = body mass index; TC = total cholesterol; HDL-C = high-density lipoprotein cholesterol; LDL-C = low-density lipoprotein cholesterol; TG = triglyceride. ^1^: Data of gender, age, geographic region, educational level, yearly income, smoking, drinking, and hypertension are expressed as *n* (%); data of BMI, energy, dietary carbohydrate, dietary fat, dietary protein, dietary fiber, dietary vitamin C, dietary vitamin E, dietary niacin, dietary selenium, TC, TG, HDL-C, and LDL-C are expressed as median.

**Table 2:** Prevalence of dyslipidemia and its subtypes (%).

Variables	Total	<50.0 mg/day	50.0–<100.0 mg/day	100.0–<150.0 mg/day	150.0–<200.0 mg/day	200.0–<250.0 mg/day	250.0–<300.0 mg/day	300.0–<350.0 mg/day	350.0–<400.0 mg/day	400.0–<450.0 mg/day	450.0–<500.0 mg/day	≥500.0 mg/day	*p*-trend *
Dyslipidemia *,‡	37.5	33.3	36.8	36.4	35.7	41.2	35.8	37.4	39.7	35.9	38.0	41.1	0.025
Hypercholesterolemia *,‡	9.6	8.0	9.6	6.7	8.8	11.1	11.3	8.5	7.3	9.0	11.3	13.6	0.004
Hypertriglyceridemia	15.2	13.3	16.8	14.1	16.1	18.7	13.2	14.8	14.2	11.7	10.0	17.8	0.863
HDL-hypocholesterolemia	21.1	19.5	22.4	23.7	20.0	22.4	18.4	22.8	22.1	19.7	20.7	20.1	0.584
LDL-hypercholesterolemia *,‡	12.7	9.6	10.1	9.4	11.8	14.6	13.4	12.4	13.0	13.9	11.3	18.3	<0.001

* Showed a significant difference in the prevalence of dyslipidemia and its four subtypes tested by Chi-square test (*p* < 0.05). ‡ Showed an uptrend tested by the Cochran-Armitage test for trend (*p* < 0.05).

**Table 3:** Adjusted ORs (95% CI) for four types of dyslipidemia across the levels of dietary cholesterol intake in adults.

Variables	<50.0 mg/day	50.0–<100.0 mg/day	100.0–<150.0 mg/day	150.0–<200.0 mg/day	200.0–<250.0 mg/day	250.0–<300.0 mg/day	300.0–<350.0 mg/day	350.0–<400.0 mg/day	400.0–<450.0 mg/day	450.0–<500.0 mg/day	≥500.0 mg/day	*p*-trend ^1^
**Dyslipidemia**												
Model 1 ^2^	0.9(0.7,1.2)	1.0(0.8,1.4)	Reference	1.0(0.8,1.3)	1.2(0.9,1.6)	1.0(0.7,1.3)	1.0(0.8,1.4)	1.1(0.8,1.5)	0.9(0.7,1.3)	1.0(0.7,1.4)	1.2(0.9,1.5)	0.451
Model 2	0.9(0.7,1.2)	1.1(0.8,1.4)	Reference	1.0(0.7,1.3)	1.2(0.9,1.6)	1.0(0.7,1.3)	1.1(0.8,1.4)	1.1(0.8,1.5)	0.9(0.7,1.3)	1.0(0.7,1.5)	1.2(0.9,1.5)	0.489
Model 3	1.0(0.7,1.3)	1.1(0.8,1.4)	Reference	1.0(0.7,1.2)	1.2(0.9,1.5)	0.9(0.7,1.2)	1.0(0.7,1.3)	1.1(0.8,1.4)	0.9(0.6,1.2)	0.9(0.6,1.4)	1.0(0.8,1.4)	0.433
Model 4	1.0(0.7,1.3)	1.1(0.8,1.5)	Reference	1.0(0.7,1.3)	1.2(0.9,1.6)	0.9(0.7,1.2)	1.0(0.8,1.4)	1.1(0.8,1.4)	0.9(0.6,1.2)	1.0(0.7,1.5)	1.0(0.8,1.4)	0.720
**Hypercholesterolemia**												
Model 1 ^2^	1.2(0.8,2.0)	1.5(1.0,2.5) *	Reference	1.4(0.9,2.2)	1.7(1.1,2.7) *	1.8(1.2,2.9) *	1.3(0.8,2.2)	1.1(0.6,1.9)	1.4(0.8,2.5)	1.8(1.0,3.3) *	2.2(1.5,3.4) *	0.066
Model 2	1.2(0.8,2.0)	1.5(1.0,2.5) *	Reference	1.4(0.9,2.2)	1.7(1.1,2.7) *	1.8(1.2,2.9) *	1.4(0.8,2.3)	1.1(0.7,1.9)	1.4(0.8,2.5)	1.8(1.0,3.3) *	2.2(1.5,3.4) *	0.072
Model 3	1.3(0.8,2.2)	1.5(1.0,2.5) *	Reference	1.3(0.8,2.2)	1.6(1.0,2.6) *	1.7(1.1,2.8) *	1.3(0.8,2.1)	1.1(0.6,1.8)	1.3(0.7,2.3)	1.7(0.9,3.1)	2.1(1.3,3.3) *	0.054
Model 4	1.3(0.8,2.1)	1.6(1.0,2.5) *	Reference	1.3(0.8,2.2)	1.6(1.0,2.6) *	1.7(1.1,2.8) *	1.3(0.8,2.2)	1.0(0.6,1.8)	1.3(0.7,2.3)	1.7(0.9,3.2)	2.0(1.3,3.3) *	0.057
**Hypertriglyceridemia**												
Model 1 ^2^	1.0(0.7,1.5)	1.3(0.9,1.9)	Reference	1.2(0.8,1.7)	1.4(0.9,2.0)	0.9(0.6,1.3)	1.1(0.7,1.6)	1.0(0.6,1.4)	0.8(0.5,1.2)	0.6(0.3,1.1)	1.2(0.9,1.7)	0.635
Model 2	1.0(0.7,1.5)	1.3(0.9,1.9)	Reference	1.2(0.8,1.7)	1.4(1.0,1.9)	0.9(0.6,1.3)	1.1(0.7,1.6)	1.0(0.7,1.5)	0.7(0.5,1.2)	0.6(0.3,1.1)	1.2(0.9,1.7)	0.597
Model 3	1.1(0.7,1.6)	1.3(0.9,1.9)	Reference	1.1(0.8,1.6)	1.3(0.9,1.8)	0.9(0.6,1.3)	1.0(0.7,1.4)	0.9(0.6,1.4)	0.7(0.4,1.1)	0.5(0.3,1.0)	1.0(0.7,1.5)	0.090
Model 4	1.1(0.7,1.6)	1.4(0.9,2.0)	Reference	1.1(0.8,1.7)	1.3(0.9,1.9)	0.9(0.6,1.3)	1.0(0.7,1.5)	0.9(0.6,1.4)	0.7(0.4,1.1)	0.6(0.3,1.1)	1.0(0.7,1.5)	0.080
**HDL-hypocholesterolemia**												
Model 1 ^2^	0.8(0.6,1.1)	0.9(0.7,1.3)	Reference	0.8(0.6,1.1)	0.9(0.7,1.3)	0.7(0.5,1.0)	0.9(0.7,1.3)	0.9(0.6,1.2)	0.7(0.5,1.1)	0.8(0.5,1.2)	0.7(0.5,1.0)	0.824
Model 2	0.8(0.6,1.1)	0.9(0.7,1.3)	Reference	0.8(0.6,1.1)	0.9(0.7,1.2)	0.7(0.5,1.0)	0.9(0.7,1.3)	0.9(0.6,1.2)	0.7(0.5,1.1)	0.8(0.5,1.2)	0.7(0.5,1.0)	0.827
Model 3	0.9(0.6,1.2)	1.0(0.7,1.3)	Reference	0.8(0.6,1.1)	0.9(0.7,1.2)	0.7(0.5,1.0)	0.9(0.6,1.2)	0.8(0.6,1.2)	0.7(0.5,1.0)	0.7(0.5,1.2)	0.7(0.5,1.0)	0.795
Model 4	0.9(0.6,1.2)	1.0(0.7,1.3)	Reference	0.8(0.6,1.1)	0.9(0.7,1.2)	0.7(0.5,1.0)	0.9(0.7,1.3)	0.8(0.6,1.2)	0.7(0.5,1.1)	0.8(0.5,1.3)	0.7(0.5,1.0)	0.514
**LDL-hypercholesterolemia**												
Model 1 ^2^	1.1(0.7,1.6)	1.1(0.7,1.7)	Reference	1.3(0.9,2.0)	1.6(1.1,2.4) *	1.5(1,2.3.0) *	1.4(0.9,2.1)	1.5(0.9,2.3)	1.6(1.0,2.6) *	1.2(0.7,2.2)	2.2(1.5,3.2) *	0.149
Model 2	1.1(0.7,1.6)	1.1(0.7,1.7)	Reference	1.3(0.9,2.0)	1.6(1.1,2.4) *	1.5(1,2.3.0) *	1.4(0.9,2.2)	1.5(0.9,2.3)	1.6(1.0,2.6) *	1.2(0.7,2.2)	2.2(1.5,3.2) *	0.160
Model 3	1.1(0.7,1.7)	1.1(0.7,1.7)	Reference	1.3(0.9,1.9)	1.5(1.0,2.3) *	1.5(1.0,2.2) *	1.3(0.9,2.1)	1.4(0.9,2.1)	1.5(0.9,2.4)	1.1(0.6,2.1)	2.0(1.3,3.0) *	0.132
Model 4	1.1(0.7,1.7)	1.1(0.7,1.8)	Reference	1.3(0.9,2.0)	1.6(1.0,2.3) *	1.5(1.0,2.2) *	1.4(0.9,2.1)	1.4(0.9,2.2)	1.5(0.9,2.5)	1.2(0.6,2.1)	2.0(1.3,3.0) *	0.177

* *p* < 0.05. ^1^: We calculated the *p*-trend by assigning median values to levels of dietary cholesterol intake and entered this variable as a continuous term in the regression models. ^2^: We adjusted multivariable logistic regression models for gender, age (18–44 years, 45–60 years), educational level (≤primary school, secondary school, ≥high school), yearly income (tertiles), geographic region (Model 1), current smoking and drinking statuses (yes, no), physical activity (continuous; Model 2), intake of energy (continuous), carbohydrate (continuous), fat (continuous), protein (continuous), fiber (continuous), vitamin C (continuous), vitamin E (continuous), niacin (continuous), selenium (continuous; Model 3), hypertension (yes, no), and BMI (continuous; Model 4).
